# Bibliographical Department

**Published:** 1880-10

**Authors:** 


### BIBLIOGRAPHICAL DEPARTMENT.

" Nothing extenuate, nor set down aught in malice."

Index Catalogue of the Library of the Surgeon General's Office, U. S. Army.—Authors and subjects, vol. I, A—— Berlinski, with a list of abbreviations of titles of periodicals indexed. Washington: Government Printing Office, 1880.

We are under obligation to Col. J. S. Billings, M. D., Surgeon U. S. Army, for a copy of the grand book of which the above is the title page. We have never attempted the notice of a medical work that gave us as much perplexity as the foregoing, as to where to begin, and then how to do justice to the great achievement; and we were almost tempted to simply quote the title page as we have done, return thanks to Col. Billings for the treasure he has bestowed upon us in the book, and then stop. We do not know now, whether such would not be the wiser course, for who is competent to say every thing that the merits of the great undertaking so richly require should be done, if spoken of at all?

The talent, the time, the labor and the means, necessary to the production of such a storehouse of wisdom and learning, must have been immense. But if all these have been necessary to its development, they have erected a monument to American genius, industry and liberality, that shall be more enduring than bronze, in this volume. That the work is without fault, and consequently beyond the pale of legitimate criticism, we do not pretend to assert, but when so much has been done, and done so wisely and so well, it closes the door against all unkind or malevolent strictures on its method or its matter.

We may venture, however, to ask Dr. Billings whether it would not be as well to mention *valuable papers* published in medical or other journals in naming the productions of authors, as to give the names of published lectures, addresses, etc., sometimes of little value or importance. We are not informed when the work will be concluded, but from its magnitude, probably several years yet will be consumed in securing its completion.

The Practitioner's Reference Book.—By Richard J. Dunglison, A. M., M. D., editor Dunglison's "Medical Dictionary," Secretary of the American Academy of Medicine, etc., etc. Second edition, revised and enlarged. Philadelphia: Lindsay and Blakiston, 1880.

We are happy to see that this valuable, we had almost said indispensable work to the busy practitioner, has so soon reached a new edition. As much as the first edition was esteemed by those familiar with its contents, we have no hesitation in expressing the conviction, that the second will be more highly prized. It contains many important additions and improvements in its contents, rendered necessary by the rapid advancement of the healing art. So numerous are these augmentations, that the second edition of the *Practitioner's Reference Book* is nearly one half larger than its predecessor.

Did space permit, we would be glad to name the new chapters that have been added, and other interesting items likewise. But we can only say to the active practitioner, purchase Dr. Dunglison's valuable book, if you would possess a "present help in time of need." The book is well gotten up and very readable.

We are idebted to the same enterprising publishers, for a neat copy of "THE SKIN IN HEALTH AND DISEASE," by L. Duncan.

Bulkley, M. D., Attending Physician for Skin and Venerial Diseases, at the New York Hospital, Out Patient Department, Late Physician to the Skin Department, Demilt Dispensary, New York, etc.

This is one of the *American Health Primers*, edited by Dr. W. W. Keen, of Philadelphia, and is one of the best we have seen. It is an interestingly written and valuable monograph, and will amply compensate any one who may peruse its contents. It should find its way into every intelligent household in the land, and be on the table of the busy practitioner also. Messrs. L. & B. have done their part in excellent style.

MYOPIA IN ITS VARIOUS PHASES, by Julian J. Chisolm, M. D., Professor of Eye and Ear Diseases, in the University of Maryland. Surgeon in charge of the Presbyterian Eye and Ear Charity Hospital, etc.

This pamphlet, which is a reprint from the Richmond, Virginia Medical Monthly, makes the serious charge, that the forced education of the growing race, threatens inroads upon the perfect sight of our young people, and that to many children, much study means annoying eye troubles in the future. The essay on myopia in its various phases, refers more especially to that form of acquired near-sightedness found among school children, and which if detected in time, can be permanently stopped. It sets out with the statement, that a growing eye is much more easily abused to its permanent detriment than the matured eye. That up to 18 years of age, the eye is acquiring firmness, and preparing for hard work. If pushed in close application during its period of growth, it may suffer serious danger by the bulging of its back wall, which bulging is most apt to occur when the eye walls offer the least resistance.

A near-sighted eye, is one which has lost its proper shape from the yielding of its walls, and the extent of disease in such an eye, is to be measured by the degree of bulging of its posterior segment on the broad principle, that the stretching and thinning of a substance does not add to its strength. The accusation that much study in young children is injurious to the eye, is not based upon hypothesis. It is the positive fruit of observation.

Specialists who have studied the eye in health and disease, have remarked that near-sightedness, as found in the schools is in a fixed ratio to the advancement of the classes, and that it was more common among children of grammar schools than among their parents. Those who have the immediate care of children, observe that during the school session near-sightedness would seemingly come on after a few months of close application, when it had not formerly excited, and would become confirmed as the education of the children progressed. From all parts of the civilized world, we have reports of the steady increase of Myopia from class to class, and from school to college, until for 4 children out of 100 in the primary schools, we find half of the graduating classes needing near-sighted glasses. Now a near-sighted eye is a diseased one, and a very near-sighted eye is one so weakened by the distension of this hollow organ, that it is liable to take on other diseases which tend to the destruction of vision. Many persons become blind in their mature age from the acquired near-sightedness of their school-life. Special observers in all countries have given us statistics which prove that near-sightedness is not only the growth of schools, but is more frequently found among those who are the closest students, especially among precocious children of tender age. The bad ventilation and crowded condition of our public schools are proverbial. The defective hygiene which belongs to all such establishments, is a prolific cause of disease, both acute and chronic. Even under the best of hygiene regulations, the overwork of a young growing eye tends to disease. How much greater is this tendency, when to bad air is added bad light; badly printed books, and so many studies as necessitate extra hours out of school to prepare them. When you add to this general crowding of book work, the additional studies of school hours, the cumulative causes of over straining tell very injuriously. The struggle for a gold medal, has injured many a girl to a degree that rendered her future life a burden. No amount of book knowledge or degree in our public schools, can compensate for the weak eyes induced by too close application.

The growing child must be educated, but at the same time while the brain is being developed, let us take also special care that a well developed body will be forthcoming to support it. Physical exercise is as necessary for future usefulness as mental development. The life of a growing child should be devoted equally between school and play, the latter preponderating in early years.

The paper which has called forth these remarks, is designed to show how the acquiring of near-sightedness of children is caused, and how it can be prevented, and also how strong eyes can be kept during the acquiring of an education.

We would recommend its careful perusal to all those who have charge of the education of the growing race. It conveys a lesson which cannot be too closely followed by teacher and pupil.

" *Ophthalmic Operations*, with remarks on after treatment; and *The Ophthalmic* use of *Quinine* and its Therapeutic Action," By A. Sibley Campbell, M. D., etc., Augusta, Georgia.

" *Suggestions on the Management* of National Labor, By J. W. Singleton, M. D., of Padnea, Ky."

" *Note on the Alkaloids of Cinchona*, By Benjamin Lee, M. D., Ph. D., F. A., A. M., of Philadelphia.

*The Medical Embassy to England*, Being a report of the trial of Bailliere V. Beaney, in the Supreme Court, Melbourne, Australia, May, 1880, with comments of the press thereon.

*Diagnosis of Malignant* Tumors of the Upper Jaw in Youth, By L. McLane Tiffany, M. D., Professor of Operative Surgery, University of Maryland, Baltimore, 1880.

" *Report of the Bureau of General Sanitary Science, Climatology and Hygiene*, to the American Institute of Homœopathy, Session of 1880, compliments of Bushrod W. James, M. D.. Philadelphia, Chairman, Philadelphia, 1880."

" *The Medical Luminary*, A Monthly Journal, Devoted to Practical

Medicine and the Collateral Sciences, New Preparations, etc., R. H. Andrews, M. D., Lansdale, Pa., Editor and Proprietor, August, 1880." Is a very neat and interesting journal.

THE TWENTIETH SESSION OF THE AMERICAN DENTAL ASSOCIATION was held at Boston, during the first week in August, 1880. There was a large attendance, and many valuable papers were read and discussed.

On Wednesday afternoon, the association accepted the courtesies of the Dentists of Boston to a drive about the suburbs of the city, and on the next afternoon, that of the Mayor and the city government, to an excursion down the harbor to a civil institution, where the inmates (children) gave a very interesting concert. We were then conducted to another chamber, where the Mayor welcomed the association with a most happy and humorous speech, and invited us to use our teeth on the sumptious repast before us.

The drive and excursion with all hospitalities, were highly enjoyed and appreciated by the members and their families, who carried away happy remembrances of the week in Boston. B. M. W.

THE JOHNSTON BROS. EXCURSION.—During the week of the several dental meetings, announced in our July issue, held in New York City, the well-known and enterprising large manufacturers of dental goods, Messrs. Johnston Bros., of 1260 Broadway, N. Y., gave an excursion on friday afternoon to the dentists and their families, up the Hudson and through N. Y. harbor, landing at the iron pier Long Island, and back to the city. While on board of their graceful little steamer, they served us with the "good things" of N. Y. market. All returned happier, and none the less grateful to these gentlemen for having the will to manufacture and give to the profession, the *first* dental engine, the *first* liquid nitrous oxide gas, the *first* American Morrison chair, and the *first pedal* lever chair ever brought before the public. B. M. W.

